# Principles of cooperation across systems: from human sharing to multicellularity and cancer

**DOI:** 10.1111/eva.12303

**Published:** 2015-10-17

**Authors:** Athena Aktipis

**Affiliations:** ^1^ Department of PsychologyCenter for Social Dynamics and ComplexityCenter for Evolution and MedicineBiodesign InstituteArizona State UniversityTempeAZUSA; ^2^ Center for Evolution and CancerUniversity of California San FranciscoSan FranciscoCAUSA

**Keywords:** cancer suppression, cheater suppression, food sharing, kinship, need‐based transfers, Walk Away

## Abstract

From cells to societies, several general principles arise again and again that facilitate cooperation and suppress conflict. In this study, I describe three general principles of cooperation and how they operate across systems including human sharing, cooperation in animal and insect societies and the massively large‐scale cooperation that occurs in our multicellular bodies. The first principle is that of Walk Away: that cooperation is enhanced when individuals can leave uncooperative partners. The second principle is that resource sharing is often based on the need of the recipient (i.e., need‐based transfers) rather than on strict account‐keeping. And the last principle is that effective scaling up of cooperation requires increasingly sophisticated and costly cheater suppression mechanisms. By comparing how these principles operate across systems, we can better understand the constraints on cooperation. This can facilitate the discovery of novel ways to enhance cooperation and suppress cheating in its many forms, from social exploitation to cancer.

## Introduction

In my work, I often think about humans and cells interchangeably – as networks of individuals sharing resources, moving and responding to various challenges with adaptive strategies. Both are capable of processing and responding to complex information in the environment by changing their behaviors and internal states. Both live in highly social environments where their fitness is interdependent with that of others. Sometimes, this interdependence is positive, and we see cooperation, for example, when individuals (whether cells or humans) rely on one another to survive (Box 1). And sometimes, this interdependence is negative, and we see conflict, like when individuals compete over limited resources. In other words, social systems can be both symphonies of cooperation and teeming masses of conflict, sometimes, at the very same time. Whether a social system ends up in a more cooperative or competitive state is a result of both the opportunities available to the actors and the decisions those actors make.

Box 1 **Hamilton's rule and need‐based transfers**

The need‐based transfer framework can be understood in the context of Hamilton's rule by integrating relative need and a broader conception of fitness interdependence (rather than just genetic relatedness). Considering Hamilton's rule (Hamilton 1964a,b), one can see that the conditions favoring resource transfers become less restrictive as relative need of the involved parties gets larger. The original formulation of Hamilton's rule can be written as follows:

$$r>\frac{c}{b}$$

where *b* is the benefit to the receiver, *c* is the cost to the giver and *r* is the relatedness between the parties. It is not necessary to know the absolute values of *c* and *b* to know the viability of the resource transfer act in Hamilton's rule. To translate Hamilton's rule to a need‐based transfer context, we can simply replace *c*/*b* with a term for relative need of the giver to the receiver:

$$r>\frac{1}{d}$$

where *d* is the relative need of the receiver compared to the giver. As relative need, *d*, increases, the conditions under which resource transfers are viable become more permissive. Even very low levels of relatedness favor resource transfers if the receiver is in much greater need than the giver. If we replace *r* with a term that denotes the fitness interdependence, shared fate or the stake individuals have in one another (Roberts 2005), *s*:

$$s>\frac{1}{d}$$

We can see that high relative need can favor resource transfers even if fitness interdependence, *s*, is low and that high fitness interdependence can favor giving even if there is little difference in the need of the recipient and giver. Interestingly, if individuals have a greater stake in another than in themselves (which may sometimes be the case for postreproductive individuals), then *s *>* *1, and givers can be favored to give even if they are more needy than the recipient. This approach also can also represent negative fitness interdependence that can occur in competitive context (*s *<* *0) including even spiteful scenarios (*b/c *>* *1/*s* and *b* and *s* are negative) (Roberts 2005).

The main purpose of this paper is to provide an overview of my work to date and provide some reflections on being a female scientist. In the following pages, I provide a brief overview of my work on the Walk Away strategy, need‐based transfers in sharing systems, and the problems inherent in scaling up cooperation to systems including human societies and multicellular bodies. I also provide some personal reflections on being a female scientist. Many of these personal reflections appear in Box 2, but throughout I have tried to provide some of the context in which my work was done and the important intellectual influences on me and on my work. I also identify what I think are some exciting new areas of inquiry in cooperation theory, evolutionary biology and cancer biology. Because the same fundamental principles underlie cooperation and conflict across systems, there are many unrealized opportunities that can be capitalized on by the next generation of interdisciplinary scientists.

Box 2 **Personal reflections**

My interest in evolution and behavior started when I was in high school. At my local bookstore I found popular books about psychology, evolution and the intersection of the two. My parents bought me book after book, many of which I read while traveling to Greece and Austria (their natal homes) during the summers between my high school years. I have many wonderful memories of reading and taking notes while being in amazing places with my parents, like the summer when my mother did charity work in a monastery in Corfu, perched above the cliffs looking over the island and the sea. Despite my father being a ‘traditional Greek’ in many ways, he encouraged my intellectual interests and career ambitions from an early age.

I arrived at Reed College at the age of 17, eager to learn as much as I could about evolution and behavior. Despite being a freshman, Mel Rutherford allowed me to take her upper‐level evolutionary psychology course my first semester and very generously supervised me in for an independent study course in evolution and behavior during the second semester. The summer after, I got an internship at the Economic Science Laboratory at the University of Arizona (U of A), where I had a wonderful (albeit hot) summer learning about experimental economics methodology. Unbeknownst to me my advisors there arranged for me to be accepted into the graduate program at U of A. Ultimately I chose to finish my undergraduate studies at Reed before beginning graduate school, a decision I do not regret. While at Reed, I had many truly wonderful professors who inspired me both in terms of my interest in the subject matter and their passion for teaching and creating a vibrant intellectual environment.

The first work I did on the Walk Away strategy was while I was still an undergraduate. At first, I thought of modeling as just a hobby: something fun to do just to explore interesting ideas. I remember sitting in a bar in Berlin with some friends – Nicole Hess who was then a graduate student and Ed Hagen who was at the time a Postdoctoral Fellow (they are now both at the University of Washington) – and telling them about my result that Walk Away could outperform tit for tat in the agent‐based model I had written. They told me that the results were interesting and that I should write it up for publication. I followed their advice and the paper that resulted (Aktipis 2004) is now my most highly cited paper.

During graduate school at the University of Pennsylvania (U Penn), I continued my work on the Walk Away strategy as well as developing several other lines of research with my graduate advisor, Robert Kurzban. I was lucky to be in a laboratory that was open‐minded to modeling approaches and to be in a graduate program that allowed me to continue this work, despite the fact that it was not the kind of work that was traditional for a psychology department. During this time I also had two children and faced the challenges of balancing my coursework, dissertation research and the care of two young children. I was very lucky to have a mentor who was a successful (and tenured) female professor who herself had several children in graduate school. She helped me to navigate the complex social and political landscape that I unintentionally created when I arrived in class, at colloquiums and lab meetings with my infant.

After completing my PhD at U Penn, I accepted a postdoctoral fellowship at the University of Arizona (U of A) in the Department of Ecology and Evolutionary Biology to work with John Pepper. At the time I still had two very young children and a husband with a job in Philadelphia. This was an important transition time in my life for many reasons. I had to make perhaps the most important ‘Walk Away’ decision of my life in terms of my marriage, and I also ended up changing the subject matter of much of my work dramatically (although using very similar methods and principles). During the two years that I commuted back and forth from Philadelphia and Tucson, I had time to reflect on my life from 30 000 feet and I made many decisions and changes. I ended up leaving my marriage and changing the focus of my research from the evolution of cooperation to the intersection of cooperation theory and cancer evolution.

My postdoctoral advisor, John Pepper, was not just the first person to introduce me to evolutionary approaches to cancer but he was also the person who introduced me to Carlo Maley, an evolutionary cancer biologist who eventually became my husband. We have perhaps the most academic love story imaginable: we fell in love while writing a grant bringing together our two fields: cancer evolution and cooperation theory. We got the grant, got married and moved to San Francisco together to start the Center for Evolution and Cancer at the University of California San Francisco (USCF). Soon after, we had a child together, for a total of three. I was lucky to have a very equal partner in parenting and excellent childcare.

From that point forward, I had to navigate the complexities of managing my own career as a young female scientist while working on a number of projects with my husband (who was senior to me and partly in the same field). Although it was rarely explicit, I sometimes encountered situations in which work that I had done collaboratively with him was treated as if it ‘did not count’ toward my record, even for projects that I had initiated and led. This is a challenge that other female scientists I know have encountered as well. We nevertheless continued to collaborate, working on papers and planning conferences on evolution and cancer during the years that we were at UCSF.

Carlo and I recently had the privilege of spending a year at the Institute for Advanced Study in Berlin (the Wissenschaftskolleg or Wiko as it is affectionately called) as part of a cancer evolution working group convened by Michael Hochberg. We had the opportunity to work together with many brilliant and fascinating scientists to identify major open questions in evolution and cancer and collaborate on several papers. One of the main outcomes of our discussions was to bring together cooperation theory with cancer evolution through considering the ways that cancer cheats multicellular cooperation. Being a part of this working group (and interacting with other working groups, especially the extreme traits working group) challenged my thinking and brought my cooperation theory work and Carlo Maley's work on cancer suppression across life together in ways that it might otherwise have taken decades. Wiko was also extremely family friendly, helping us arrange schools, babysitters, and even providing free babysitting every Thursday for the fellows’ dinner. It is experiences like that year at Wiko that make being an ‘academic couple’ worth all the complexities that it brings.

Even systems made up of seemingly identical entities cooperating for a common goal, for example, cells in a multicellular body require suppression of conflict. Cell‐level cheating is just suppressed strongly enough and long enough for the multicellular body to survive and function effectively until reproductive age (Brown and Aktipis 2015). All large‐scale cooperative systems, even those made up of genetically very similar or identical entities, require redundant layers of checks and balances to suppress the conflict that would otherwise undermine the stability of the system.

The simultaneous existence of cooperation and conflict in large‐scale systems can be understood in terms of fitness interdependence: in domains where fitness is positively interdependent, there are cooperative opportunities, and in domains where fitness is negatively interdependent, there are challenges that arise from conflict (Roberts 2005). Cooperation and conflict can and often do exist simultaneously in any sufficiently large, complex, and long‐lived cooperative system, whether it is a multicellular body, social insect colony, or human group (Strassmann and Queller 2010).

The level at which selection is operating greatly influences the strategies that will evolve in these systems. Individual selection favors strategies that prioritize individual survival and reproduction, as is the case with cancerous cells evolving in the body or organisms evolving in a highly competitive environment. Higher levels of selection (including what have been called group selection and multilevel selection) favor strategies that enhance survival and reproduction of the larger kin group, aggregation, or network. Fitness interdependence of the individuals making up these groups may help to predict the relative power of selection operating at these different levels (Roberts 2005).

In most systems, selection operates simultaneously at many different levels of organization. Take humans, for example: selection simultaneously operates on the organism‐level favoring cancer suppression mechanisms and at the cell‐level favoring neoplastic cells (Aktipis and Nesse 2013). It also operates on the level of genes and chromosomes, with genetic conflict being actively suppressed within our genomes to prevent the spread of selfish elements (Hurst et al. 1996). Selection can also operate on the level of kin groups or other aggregations, favoring more cooperative partnerships and groups, as long as the structure of the population promotes sufficient positive assortment (i.e., preferential interactions of cooperators with one another). If positive assortment is high then individual‐level selection favoring exploiters do not undermine selection for cooperation at higher levels of organization (Eshel and Cavalli‐Sforza 1982; Pepper and Smuts 2002; Fletcher and Doebeli 2009).

Selection at competing levels has shaped behavior and physiology of many different systems. Selection among individuals has led to effective competitive behavior, cheater detection, and the ability to respond effectively to the environment in many ways (including moving, changing state, and altering the environment). Selection at higher levels is important as well, whether we want to call it group selection, multilevel selection, or social selection. These higher levels of selection are what favored the evolution of multicellularity and probably also what favored many aspects of human sociality that we see as fundamental to our humanity: our willingness to cooperate, our capacity to entrain with one another, and our concern for the well‐being of others, whether our genetic kin or others with whom we have interdependent fates.

These examples make the questions of ‘what level of selection is most important?’ seem somewhat misguided (Kurzban and Aktipis 2007). Selection is operating on multiple scales simultaneously and it is the interaction between these scales that shapes the cooperation and conflict that we see at every level. My work spans many different scales and systems so I am constantly reminded of the ways in which selection for competition at one level can be suppressed by selection for cooperation at a higher level. In the case of cancer suppression, this works partially through organism‐level suppression of evolution itself: tissue architecture and the constraints on cell proliferation (including having only a subset of stem‐like cells capable of proliferating) are in place largely to prevent somatic evolution that could lead to cancer (Cairns 1975). In humans many different cheater suppression mechanisms contribute to our capacity to cooperate effectively, some of which are external such as our genetic similarity to others or our embeddedness in social systems with institutions and norms. Others may be internally regulated, like or feelings of commitment or conscience. And yet other cheater suppression systems may be cocreated with our social groups, making them potentially unique and diverse across cultures (and subject to cultural evolution). Evolution can operate on all of these cheater suppression systems at multiple levels of organization, from small groups to large social organizations.

## Overview of scientific contributions

As a freshman at Reed College, I took Mel Rutherford's Evolutionary Psychology course, getting my first academic introduction to evolution and behavior. Dr. Rutherford was a visiting professor who had studied with Cosmides and Tooby (two of the leading figures in the field of evolutionary psychology) at the University of California, Santa Barbara. Through this course, I read Axelrod's seminal work on the evolution of cooperation (Axelrod 1984) and came to realize that computational approaches had the power to answer many important questions about cooperation in humans and in other systems. I was so intrigued by the prospect of addressing these questions computationally that I decided to teach myself how to program, first in C++ and Java, and then agent‐based modeling platforms that easily enabled the inclusion of space and individual decision‐making rules. I was particularly drawn to the question of whether the ability of individuals to leave interactions with defectors could stabilize the evolution of cooperation. Earlier work on mobility and the evolution of cooperation showed that movement provided an advantage for defectors (Dugatkin 1992; Dugatkin and Wilson 1992; Ferriere and Michod 1995, 1996). I was curious whether conditional movement (as opposed to random movement) could provide an advantage for cooperators by allowing them to preferentially interact with one another and avoid continued interactions with defectors.

### Know when to Walk Away

Individuals of many types Walk Away from bad situations: humans leave bad relationships, foraging animals leave exploited patches, and cancer cells leave hypoxic (low oxygen) environments. It is easy to see how such a rule would evolve: individuals who leave bad environments have higher fitness than those who stay. But what happens when groups of individuals use this rule to respond to their social environments is something quite astounding: the cooperative option comes to have higher fitness than defection and that enables cooperation to take over a population of defectors.

**Figure 1:**
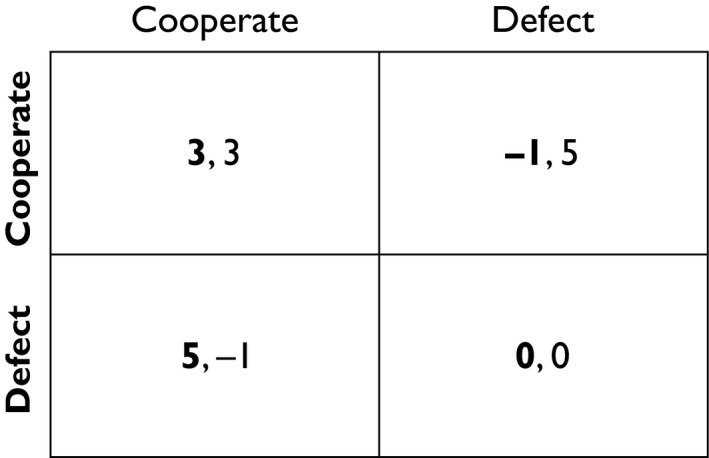
Payoff matrix for the Prisoner's Dilemma. Row player is in bold.

**Figure 2:**
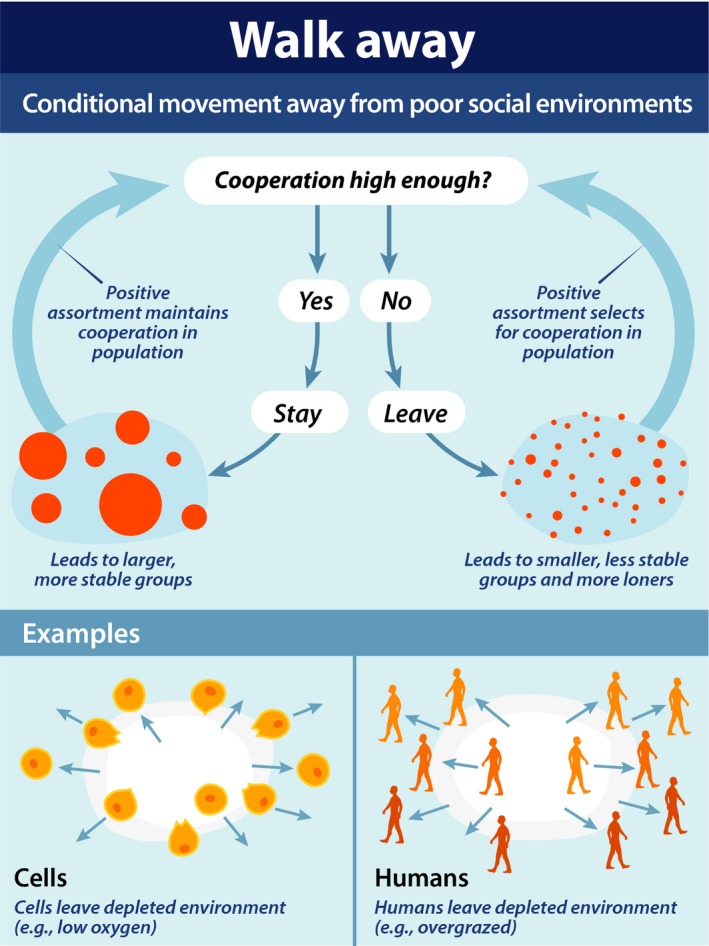
The top panel shows the basic Walk Away rule, with individuals conditionally leaving uncooperative groups. When individuals use such a Walk Away strategy, this increases the stability of cooperative partnerships and groups, increasing positive assortment (i.e., the likelihood that cooperators will interact with one another relative to defectors). If cooperation is initially low, positive assortment leads to selection for cooperation (right side loop); if cooperation is initially high, positive assortment helps to maintain it (left side loop). The bottom panels give two examples of Walk Away in the natural world: cells leaving low oxygen (hypoxic) regions and humans leaving depleted environments.

The Walk Away strategy (and conditional movement more generally) is fundamentally very simple. In fact, Walk Away is a social generalization of a foraging rule that states ‘stay if things are good, otherwise leave’. It breaks the frame of the traditional prisoner's dilemma game (Fig. 1) in that individuals have the ability to leave if they are not satisfied with the payoffs from being a part of their current partnership or group. This fits with many real world situations that humans, cells and other organisms experience where they have the ability to leave an interaction or environment that is not favorable (with, of course, some important exceptions which I will address later). The basic features of the Walk Away strategy and its implications for cooperation in various systems are illustrated in Fig. 2.

When I went to graduate school to work with Rob Kurzban at the University of Pennsylvania (U Penn), I continued working on models of Walk Away and other cooperation topics. It was in graduate school that I had the chance to fully develop these models and begin turning them into successful publications with the advice and mentoring of my advisor, Dr. Robert Kurzban.

The two‐player Walk Away model is based on the prisoner's dilemma payoffs (Fig. 1), in which two agents each decide whether to cooperate or defect in a given round of play. Walk Away agents (i.e., agents that have the ability to leave after a partner defects) play against traditional tit‐for‐tat players that copy the previous partner's behavior and several other strategies. In these models, cooperative Walk Away agents outperformed all other strategies that were included in the simulation (Aktipis 2004). This means that a strategy of All‐Cooperate is viable as long as cooperators have the ability to ‘Walk Away’, that is, leave defecting partners. Cooperators do not need to have the ability to avoid defectors, only the ability to respond to the fact that they interacted with a defector by moving to another location in space. This is different from Enquist and Leimar (1993) proposition that cooperation in mobile organisms requires antifree riding adaptations beyond mobility itself. The results of the Walk Away model are similar in some ways to Enquist and Leimar (1993) results: both models found that defectors only outperformed cooperators when search costs for new partners were low. However, Enquist and Leimar concluded that mobility restricts cooperation, while the Walk Away model demonstrates that conditional movement can create the conditions that select for cooperation. These different conclusions are likely a result of both differences in the modeling approach (agent‐based versus analytical) and different rules for ending pair‐wise interactions. In the Walk Away model, partners stayed together until one partner left or the pair was disrupted, while in Enquist and Leimar's model pairs stayed together until one partner left *or* when the maximum coalition time was reached (Enquist and Leimar 1993).

**Figure 3:**
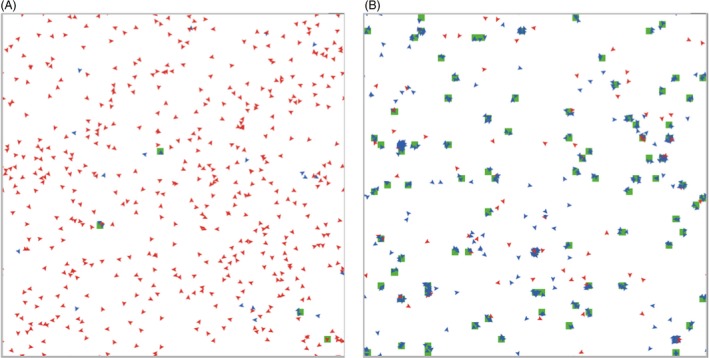
Screenshots from the group‐wise Walk Away model (Aktipis 2011). (A) At the beginning of the model (*t* = 200), defectors (red) dominate the population. (B) Later, (*t *=* *40 000) cooperators (blue) are more common, and groups are larger, more productive (as indicated by green patches) and more stable.

In my work, I have also implemented the Walk Away strategy in a group‐wise context, where agents have the opportunity to invest in a public good, and the results of that investment are distributed evenly to all group members regardless of who invested (Aktipis 2011). This is analogous to a situation like a work team where individuals might vary in their contributions, but every team member gets equal credit for the outcome. In the model, individuals are either cooperators who invest in the public good or defectors who do not. Every individual has a threshold for the level of cooperation required to stay in their current group, else they leave and move around randomly until they encounter a new group. This leads to cooperative groups being more stable and uncooperative groups being less so (Fig. 2), which leads cooperative individuals to have more opportunities to interact and receive cooperative payoffs. The group structure emerges from agents staying, leaving, and the successful agents reproducing. Groups are not predefined as they typically are in public goods models. In the Walk Away model, the structure and dynamics of the population change over time, initially being dominated by migrating defectors (Fig. 3A) and later stable cooperative groups (Fig. 3B). Walk Away cooperators outperformed defectors over most of the parameter space investigated. This was the case as long as Walk Away agents were somewhat intolerant of defectors, not staying in groups composed of more than half defectors. Even when some defection was tolerated, cooperators were able to preferentially assort as a result of this very simple movement rule and that assortment allowed cooperation to evolve and be stable.

Walk Away favors cooperation because it promotes positive assortment, the preferential interactions of cooperators with one another. Positive assortment has been proposed as the fundamental principle underlying the evolution of cooperation across systems (Fletcher and Doebeli 2009). When cooperative individuals are able to preferentially provide benefits to other cooperators and limit interactions with defectors, cooperation becomes much more evolutionarily viable. This basic principle drives the evolution of cooperation via kin selection, group selection, reciprocity and virtually every explanation of cooperation that has been given in the cooperation theory literature to date (Fletcher and Doebeli 2009). This means that the Walk Away rule can promote the evolution of cooperation in many different contexts, including both interactions among kin and nonkin.

The effectiveness of conditional movement strategies in promoting the evolution of cooperation via assortment has been shown in a number of other models. These include Pepper's environmental feedback models, which simulate strategies based on simple foraging rules and show that the cooperative trait of restrained eating behavior can evolve (Pepper and Smuts 2002; Pepper 2007) and a nonspatial conditional movement model in which individuals are automatically re‐paired with a new partner after leaving (Schuessler 1989). A model published soon after the original Walk Away article demonstrated that conditional movement away from defectors can evolve when agents have a broad potential behavioral repertoire (Hamilton and Taborsky 2005), and a more recent study showed that conditional mobility is more effective than conditional cooperation (Izquierdo et al. 2010). Empirical work also shows that conditional movement enhances assortment and may therefore play a central role in the evolution of prosocial behavior. For example, in water striders, female ‘Walk Away’ behavior appears to limit the success of aggressive males, leading selection to favor less aggressive males that would otherwise be the case (Eldakar et al. 2010). Studies in the laboratory with humans have also found ‘Walk Away’ behavior when individuals have the option to exit the interaction after an encounter with a defector (Orbell et al. 1984).

The Walk Away rule and conditional movement more generally belong to the class of partner‐choice rules. These rules can favor cooperation by creating implicit or explicit competition for being a good partner in biological or social ‘markets’ (Noe and Hammerstein 1994; Barclay 2013). Partner choice can favor the evolution of cooperation through social selection, a process parallel to sexual selection in which costly traits may be favored if they increase the likelihood of being chosen as a social partner by other cooperators (West‐Eberhard 1979; Nesse 2009). In partner‐choice models, individuals have the ability to determine who they will and will not interact with based on information they have gathered from past behavior or interactions. This enables cooperators to preferentially interact with each other, which promotes behavioral assortment and favors cooperation (Fletcher and Doebeli 2009).

Walk Away is an extremely simple partner‐choice rule as it requires no memory, reputation, or other complex information‐processing capabilities. This is in contrast to some complex models of conditionally moving agents that came before Walk Away (e.g., Vanberg and Congleton 1992; Yamagishi et al. 1994). Walk Away simply responds to the current payoffs it receives, leaving partners or groups that are insufficiently cooperative. This rule is effective despite its simplicity because it limits the costs to cooperators of interacting with noncooperators and it changes the dynamic structure of the population in ways that favor cooperation (Figs 2 and 3). This simplicity is one of the strengths of Walk Away as a model for the evolution of cooperation across forms of life including those without nervous systems. However, this simplicity is also a limitation when it comes to understanding more complex forms of cooperation, including those that characterize human social living.

In our lives and careers and relationships, we do not always have the ability to decide when to stay and when to leave. Some of these constraints on the ability to Walk Away are a result of our own previous actions. For example, if we have invested a lot in a shared goal or endeavor, we might experience of feeling of ‘sunk costs,’ not wanting to give up on something that we have already invested so much in. We might also commit emotionally to a person or group, inhibiting our Walk Away behavior. Other inhibition of Walking Away could be because of fear of the consequences of leaving, either because there are no good outside options or because of the threat of punishment.

Many of these constraints are instantiated by norms, institutions, and culture. Some of them probably help to enhance cooperation and prevent exploitation, but others may be in place to enable some individuals to exploit others. We participate in many long‐term and investment‐intensive activities that require long‐term associations, commitment, and sometimes even signaling that we have cut off outside options. We also have the ability to bond with each other, which constrains our willingness to leave if things are not good. Many signals of commitment are basically ways of saying ‘I will never Walk Away’. My work on the Walk Away strategy has so far been limited to modeling Walk Away in dyads playing the prisoner's dilemma (where it outperforms both tit for tat and defection) (Aktipis 2004) and in public goods games (where cooperators dominate the population) (Aktipis 2011). But extending this to specific questions about human relationships requires taking a broader perspective.

Humans probably have a Walk Away‐like rule, but it almost certainly operates on a longer time scale than just considering the payoffs of the most recent interaction. Also, the question of how leaving thresholds are set (i.e., how much will you tolerate being exploited before leaving?) is an important open question. Previous experience might influence the leaving threshold and the time frame over which previous payoffs are integrated when considering whether or not to leave. In humans, our willingness to Walk Away is probably often heavily influenced by our emotional engagement and bonds with one another.

I would offer that Walk Away, or the ability to leave a bad social situation, is in some ways the ‘ancestral’ state, with other emotional capacities such as bonding building upon it and modifying it in various ways to allow us to benefit more from long‐term associations and signal our cooperative intentions. Future work could extend the Walk Away model to investigate the specific questions about human relationships, including how bonding and attachment may inhibit Walk Away behavior and alter selection on the evolution of cooperative and defecting strategies. In human relationships (and likely in other systems), information over multiple time periods and from multiple sources is likely to be included in the decision to Walk Away or stay including the assessment of the value of outside options and the costs associated with leaving. Future Walk Away models may help us answer questions about human sociality by incorporating these factors.

### Taking an evolutionary approach to cancer

My postdoctoral advisor, John Pepper, was the first person who introduced me to evolution and cancer. I came to the University of Arizona (U of A) to work with him on models of organismal mobility and cooperation since he had worked extensively on that topic (Pepper and Smuts 2001, 2002; Pepper 2007), but we soon realized that the evolution of motility was likely to be occurring in cancer, with potentially important implications for cancer progression. We decided to develop a model of dispersal evolution in neoplasms to test whether cells that exploited resources evolved to be more mobile. Our model showed that high cell metabolism leads to the evolution of higher cell motility (Aktipis et al. 2012). More generally, our model and other models suggest that dispersal evolution may play an important role in invasion and metastasis in cancer (Chen et al. 2011; Aktipis et al. 2012).

Walk Away dynamics may also help to illuminate other aspects of cancer biology that are poorly understood. For example, Walk Away processes such as cells leaving hypoxic conditions or poorly maintained microenvironments could contribute to the dynamics of invasion and metastasis (Schiffman et al. in press). Cells are able to engage in Walk Away behavior through upregulating motility factors when in poor environments (Pennacchietti et al. 2003) (Fig. 2). This means that early microenvironmental destruction could contribute to initial invasion in cancer.

During the time I spent at U of A as a postdoc, I also had the privilege of spending time in the laboratory of Rick Michod, who has worked extensively on the evolution of multicellularity from a cooperation theory perspective (e.g., Michod 1999; Michod and Roze 2001). I learned a great deal from the time that I spent in that laboratory, especially interacting with Aurora Nedulcu (who was visiting from the University of New Brunswick at the time). As a result of all of these interactions in Dr. Pepper's and Dr. Michod's laboratory, I realized that there were many important connections between cooperation, multicellularity, and cancer.

I also realized that the Walk Away model could be extended to explore questions about the evolution of multicellularity: the first multicellular entities may simply have been dividing cells that ‘decided’ not to Walk Away from each other despite resource competition, perhaps because there were public goods being produced that made staying have a higher payoff than leaving. Later, other regulatory systems are likely to have evolved that modified this Walk Away behavior to make multicellularity more viable. These are open questions that arise from the Walk Away framework and could have important implications for our understanding of the evolution of both multicellularity and human sociality.

### Giving to those in need

Resource sharing is feature of cooperation across many different kinds of systems at a variety of scales. From hunter–gatherers engaging in what anthropologists call ‘central place food sharing’ to multicellular bodies transporting resources to cells that need them, cooperative systems are characterized by what my colleagues and I have termed ‘need‐based transfers.^1^’ Need‐based transfers are exactly what they sound like: resource transfers that are conditional on the need of the recipient (and usually also the ability to give).

**Figure 4:**
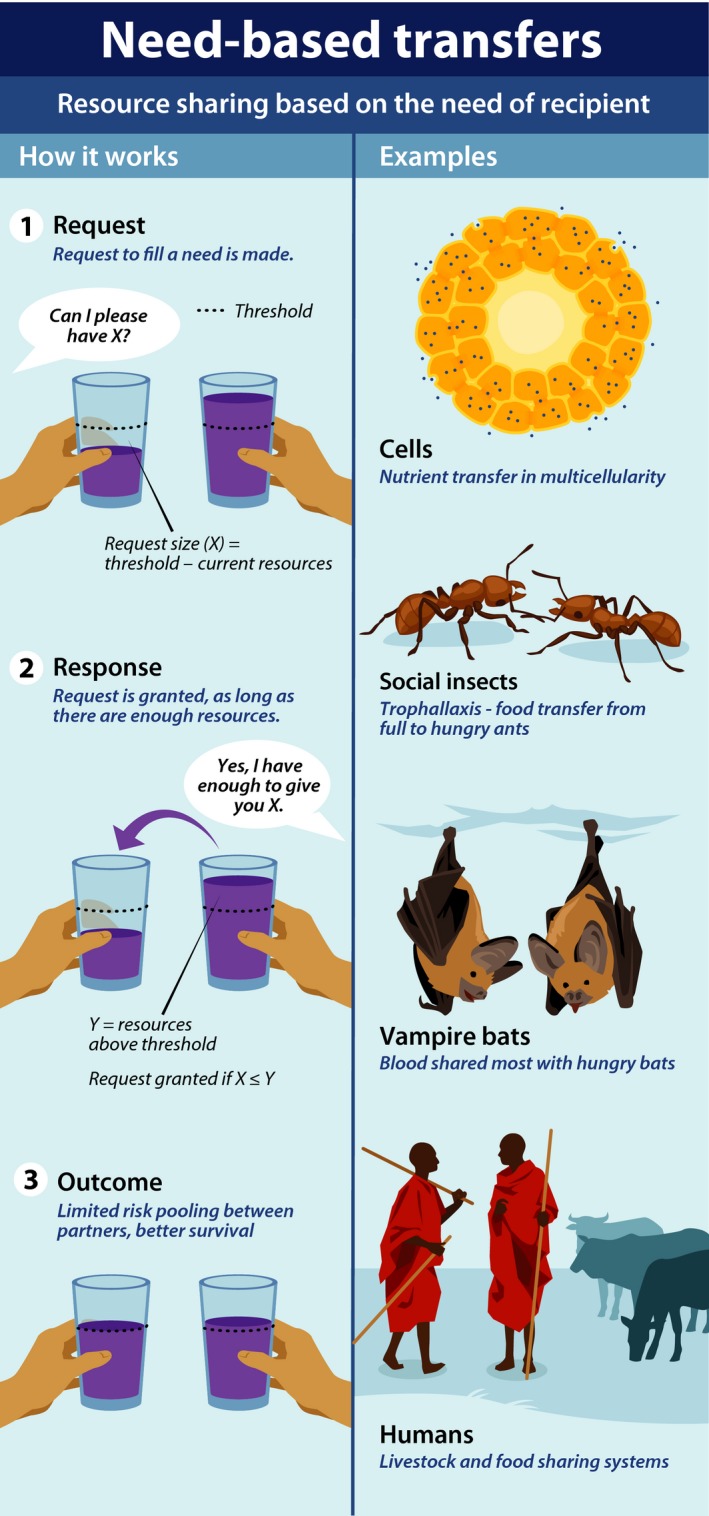
This figure illustrates the basic components of need‐based transfer systems and some examples. The left panel shows basic steps in need‐based transfers: first a request is made, then a response is made in the form of a resource transfer (assuming certain conditions are met, which can lead to positive outcomes in the form of greater risk pooling and higher survival. The right panel provides four examples of need‐based transfers in biology: cells sharing resources, ants transferring food through trophallaxis, vampire bats sharing blood meals, and humans sharing food or livestock.

There are many other systems in which need‐based transfers occur, both human and nonhuman (Fig. 4). According to the ethnographic literature, and our preliminary fieldwork at several of our sites, many small‐scale societies around the world engage in resource sharing based on the need of the recipient (L. Cronk and C. A. Aktipis, unpublished data). Many nonhuman species use need‐based transfers to cope with risky environments and uncertain resource availability. Vampire bats famously studied for their apparently reciprocal food sharing were also engaging in need‐based transfers, with bats in dire need being the most likely to receive blood from donors (Wilkinson 1984). Ants and other social insects engage in a food‐sharing behavior called trophallaxis. An ant coming back from successful foraging will return to the nest searching for nest mates with whom to share. Recipients may simply tap the donor's body lightly on the antenna or forelegs, inducing the donor to orient toward the recipient and regurgitate a droplet of food (Hölldobler and Wilson 2009). These behaviors of bats and ants are parallel to what we often see in human need‐based transfer systems: an individual in need makes a request and the donor gives when he/she has a resource surplus. Similar need‐based rules also operate among cells making up multicellular bodies and are likely to have been central to the evolution of large multicellular forms of life (Knoll 2011).

The need‐based transfer framework for cooperation is a formalization of two simple resource transfer rules (1) ask only if in need and (2) give if you are asked and able (Fig. 4). Like reciprocity, it can involve a special relationship between two parties, but unlike strict account‐keeping style reciprocity, it does not involve tracking of debt and credit. The evolutionary logic for need‐based transfers can be understood through extension of Hamilton's rule (Hamilton 1964a,b), where relative need affects the costs and benefits of resource transfers among parties, and the relatedness term is generalized to include all forms of fitness interdependence (Box 1). If the relative need of the recipient is high, then resource transfers can be viable even with very low levels of relatedness or other forms of fitness interdependence. If one individual in a pair is very much in need and the other individual has more than they need to survive, then the cost, *c*, to the wealthy individual of giving to the needy individual is very low and the benefit, *b*, to the needy individual is very high. The importance of relative need in the evolution of cooperation has long been recognized in the context of reciprocity (Boyd 1992). The need‐based transfer framework highlights the importance of relative need in the viability of cooperation and calls attention to the fact that *c* and *b* can vary in behavioral time given different condition of the individuals and the threats and opportunities present in their environments.

The need‐based transfer framework also has important connections to signaling theory as requests associated with need are typically signals sent from one individual to another. John Maynard Smith described the Sir Phillip Sidney Game (Smith and Harper 2003), which is essentially a need‐based transfer game. Its name is drawn from the purportedly true story of Sir Phillip Sidney who was fatally wounded on the battlefield and gave his water to another solider saying ‘thy necessity is greater than mine’. In the Sir Phillip Sidney Game, there are two individuals, a signaler who can be in one of two states, either in need or healthy, and a responder who can either give (at some cost) or not give. Both players are then assumed to act to maximize their inclusive fitness. This models shows that signaling of need can evolve among relatives (Smith and Harper 2003), suggesting that need‐based transfers may have initially evolved in the context of parental investment. Subsequent work suggests that the conditions favoring honest signaling and giving may be more restrictive than initially thought (Bergstrom and Lachmann 1997, 1998). Future work explicitly linking need‐based transfers and the evolution of signaling of need may help to answer open questions about the information conditions under which signals of need and need‐based resource transfers can evolve.

I first became interested in need‐based transfers in the context of the Maasai gift giving systems known as ‘osotua,’ which I learned about from Lee Cronk. Before beginning my postdoctoral work at U of A, I had contacted Dr. Cronk about doing a postdoctoral fellowship with him. He had no positions available, but wrote me back with an idea for an agent‐based model. He described the osotua systems of the Maasai, where individuals form special relationships with one another that they can call upon in times of need. Dr. Cronk wanted to develop an agent‐based model together to test whether this system provided benefits for the individuals using it. I was intrigued by deceptively simple osotua rules: ask only if in need, give if you are asked and able. I wanted to know whether such a simple rule could enhance survival, and so Dr. Cronk and I began what has grown into a decade‐long collaboration examining need‐based transfers, first in the context of the osotua system and then expanding to many other societies and systems.

The osotua system is akin to the kinds of relationships that many of us have with old friends that we know we can depend on, even if we have not seen them for years. In the Maasai system, this often takes the form of requests for cattle and other livestock after an unexpected event such as drought, disease, or theft.

Cheating in need‐based transfer systems is different from cheating in account‐keeping reciprocity systems (where rules typically boil down to not taking benefits without incurring costs). In need‐based transfer systems, the rules are simple: ask only when in need and give if asked and able. Cheating in this system is then parallel to that: asking when not in need and not giving if asked and able. This means that very little information is needed to determine whether somebody has cheated in a need‐based transfer system. One only needs to know the size of the request and the resource holdings of the giver and receiver. You might now be wondering how a system like this avoids a situation where one individual repeatedly asks another for resources and never gets paid back. Interestingly, it does not. If one individual is wealthier and luckier than the other round after round or year after year, resources may flow largely one way in the direction of need. This is very different from the rules of engagement in account‐keeping reciprocity systems.

This is not to say that need‐based transfer systems are characterized by exploitation. Among the Maasai, there are ‘3Rs’ that come along with osotua: restraint, respect, and responsibility. Maasai herders are expected to be restrained in their use of resources, respectful of others (especially those on whom them depend) and responsible in the care of their herd. These 3Rs help to solve the moral hazard problem that can result when individuals do not carry all the risk associated with their behavior by requiring that everyone will behave in ways that reduce the likelihood of a negative event.

The initial model and the paper that came out of our early work together (Aktipis et al. 2011) began as an interesting side project for us both. During the early years of our collaboration, I finished my PhD and began a postdoc, and Dr. Cronk was working on a book about cooperation and coordination (Cronk and Leech 2013). Nevertheless, we met about once a month, often on the weekends, and the project slowly grew and grew. Our original need‐based transfer model showed that the osotua system helps Maasai herders to pool the risk associated with living in an uncertain environment (Aktipis et al. 2011). Since then we have extended and expanded this model to examine need‐based transfers in networks of individuals. We found that larger more connected networks provide greater risk pooling, although with decreasing marginal returns after networks are sufficiently large and well connected (Hao et al. 2015).

We also discovered that need‐based transfer rules can outperform account‐keeping and that the payoffs for each of these strategies correspond to a stag hunt or coordination game. This means that both parties do better if they use the same strategy rather than each using different strategies. It also means that either strategy can be stable and that there is no incentive to switch from one strategy to another unless the parties can coordinate to both switch to the higher payoff coordination point (which happens to be the need‐based transfer strategy) (C. A. Aktipis, R. DeAguiar and L. Cronk, unpublished data). We have also found that need‐based transfer systems lead to less wealth inequality than is the case with no transfers (Hao et al. 2014) or with account‐keeping rules (C. A. Aktipis, R. DeAguiar and L. Cronk, unpublished data).

Our work also suggests that the osotua system, and need‐based transfer more generally, can effectively scale up to larger networks (Hao et al. 2015). However, many important questions remain about the viability and comparative performance of need‐based transfer systems relative to account‐keeping in the presence of cheaters. We are now investigating the question of cheating in need‐based transfers through both computational modeling and ‘cheater detection’ experiments with human subjects. Early results of these studies suggest that humans may have specialized reasoning systems for detecting cheating in need‐based transfer rules (Chang et al. 2015).

Our work on need‐based transfers has now grown into a much larger and more interesting project than we could have anticipated. We now have a multidisciplinary research project, The Human Generosity Project (www.humangenerosity.org), which is funded largely by the John Templeton Foundation and the National Science Foundation. This support has allowed us to assemble a large team to examine need‐based transfers across societies. Our methods include fieldwork at seven field sites around the world, human subjects experiments, computational modeling, educational initiatives in collaboration with the Exploratorium science museum in San Francisco, and outreach efforts with policymakers to examine the viability of need‐based transfers for solving modern resource management challenges.

In addition, my laboratory is now exploring the implications of need‐based transfers for multicellularity and cancer. Cells in our multicellular bodies are constantly redistributing resources, a process necessary for keeping our bodies alive and healthy. Need‐based transfers are likely to have played a central role in the evolution of multicellularity given the necessity of transporting resources for the growth of multicellular aggregations beyond a few millimeters in diameter (Knoll 2011). As aggregations of cells became larger, they required transport systems to move resources from the cells on the outside to those on the inside that could not get sufficient oxygen and nutrients from diffusion (Beaumont 2009). In fact, models show that resource sharing can make larger aggregations of cells more fit than smaller ones (Pfeiffer and Bonhoeffer 2003), suggesting that need‐based transfers could have contributed to the viability and competitiveness of larger aggregations over smaller ones.

Need‐based transfers among cells form the backbone of resource transport in multicellular bodies. This is the case in simple multicellularity where junctions between cells allow resources to flow to cells in regions with lower resources, and it is also the case with more complex forms of multicellularity that have bulk transport systems such as our circulatory system (Knoll 2011). Our circulatory systems’ primary function is to transport resources to peripheral tissues. It is also able to dynamically respond to the signaling of cells in low resource conditions that release signals (called angiogenic signals) that lead to the growth of new blood vessels to bring resources to cells signaling need, a process that is often exploited by cancer cells (Hirota and Semenza 2006). If cancer cells upregulate angiogenic signaling and/or increase their rate of resource use, this can contribute to cancer (Aktipis et al. 2012). Further, the ability to take up more resources can lead to deterioration of the shared environment and select for cell dispersal and metastatic capacity (Aktipis et al. 2012; Schiffman et al. in press). Thus, it appears that the framework of need‐based transfers and cheating may have important implications for our understanding of cancer progression.

All in all, the need‐based transfer framework has been a productive tool that has led to many models and novel hypotheses. Through The Human Generosity Project, our team is using results from our models in conjunctions with fieldwork and human subject experiments to better understand the decision‐making rules underlying need‐based transfers, how they manifest across societies, and the way that they scale up to larger networks of interacting individuals in many diverse systems.

### Challenges in cheater suppression

**Figure 5:**
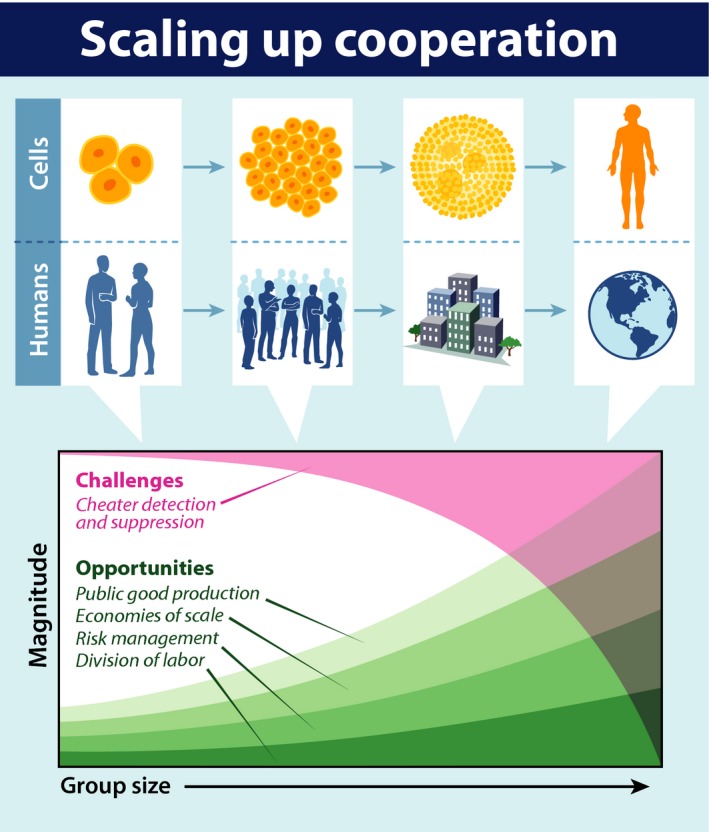
Scaling up cooperation from small‐scale social interactions to large‐scale societies presents similar opportunities and challenges across systems, from the evolution of multicellularity to human groups. Larger cooperative groups present opportunities for enhanced public good production, economies of scale, risk management, and division of labor. However, larger groups also have greater challenges when it comes to detecting and suppression cheaters.

When cooperation shifts from small‐scale interactions to massively large‐scale cooperation of the kind that we see in human societies, eusocial insects and multicellular bodies, new challenges and opportunities arise (Fig. 5). Larger scale social interactions offer many opportunities: individuals can insulate themselves from risk through resource sharing and achieve greater overall productivity through division of labor and even benefit from what economists call ‘economies of scale’— the ability to get higher marginal returns with larger scale operations (up to a certain point). Scaling up also presents with a number of new challenges including resource distribution challenges and the creation and management of a shared environment (e.g., removing waste), and the necessity of regulating reproduction and in some cases death (e.g., in the case of apoptosis in cells).

If individuals do not solve these problems effectively, then large aggregations of individuals become unstable and evolutionarily inviable. For example, a deteriorating shared environment can lead individuals to leave (i.e., Walk Away). We see this among cancer cells with the upregulation of movement factors in hypoxic conditions (Pennacchietti et al. 2003) and in foraging and dispersal behavior in many species (Stephens and Krebs 1986; Dieckmann 1999). Another example of a failure to solve the problem of cheating effectively is in the regulation of proliferation. In aggregations in which there is an optimal group size based on resource constraints, a failure to properly regulate proliferation (and/or death) can lead to overgrowth, threatening the viability of the whole aggregation.

**Figure 6:**
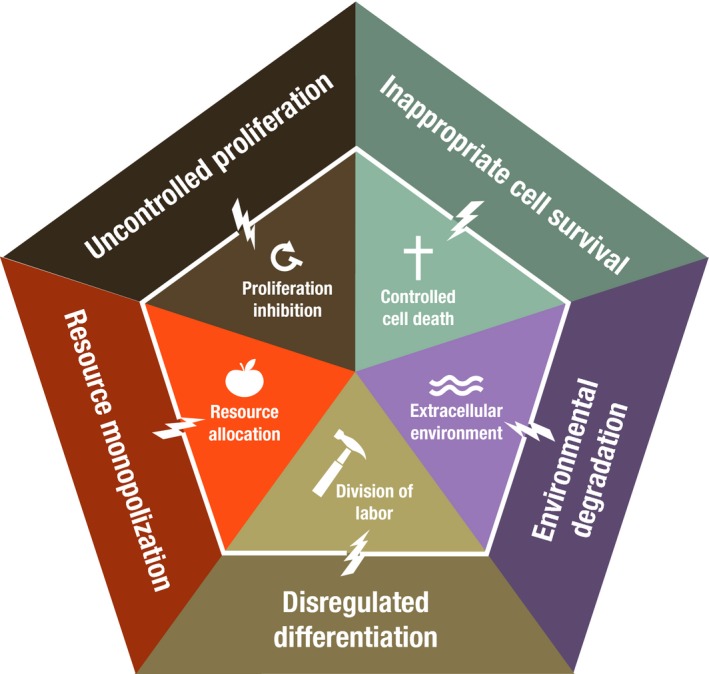
The five foundations of multicellular cooperation (center) are proliferation inhibition, controlled cell death, resource allocation, division of labor, and maintenance of the extracellular environment. When these foundations break down (represented by the lightning bolts), the outcome is cancer or cancer‐like phenomena (outside of pentagon). This figure is reprinted from Cancer Across the Tree of Life (Aktipis et al. 2015).

The challenges and opportunities associated with scaling up cooperation can clearly be seen in the evolution of multicellularity and cancer. Multicellularity requires evolving the capacities for cooperation and suppressing cheating in what we have termed the five foundations of multicellularity: proliferation inhibition, control of cell death, resource allocation, division of labor, and extracellular environment maintenance (Aktipis et al. 2015). We found that a breakdown of each of these forms of cooperation was associated with cancer and cancer‐like phenomena across species (Fig. 6). We also found that cheating in these foundations corresponded to the hallmarks of cancer (Hanahan and Weinberg 2000, 2011).

In the case of multicellular bodies and eusocial insects, selection has not only favored large‐scale cooperation and cheater suppression, but it has also resulted in the ability to reproduce at the aggregate level. Multicellular organisms produce offspring that undergo a multicellular development program as they grow, and eusocial insect colonies create new colonies that go through a kind of superorganism developmental program and grow to full‐size colonies. The ability to reproduce as an organism or superorganism is often considered an important component of what defines an organism or individual (Michod 1999; Strassmann and Queller 2010).

To reach this new level of individuality, the component cells or organisms must effectively suppress cheating and commit to cooperation (Michod and Roze 2001; Strassmann and Queller 2010). How does this commitment happen? In the case of multicellular bodies, the developmental program for the whole organism commits some cells to be dead‐end somatic cells and others to be germ‐line cells that propagate the genes of the multicellular organism into the next generation. However, somatic cheating often emerges despite these controls and so multicellular bodies like ours have backup systems: inappropriately proliferating cells are targeted for immune destruction (although cells that can evade the immune system can sometimes continue proliferating, which can lead to cancer). Social insect colonies have similar systems; with different castes have different gene expression states that commit them to nonreproductive functions. But they too often have backup policing systems where any eggs laid not by the queen are detected and destroyed. Interestingly, committing to reproducing as a unit limits the ability of cells to ‘Walk Away.’ Perhaps these complex and multilayered cheater suppression systems are necessary to make cooperation viable in these systems because Walking Away (and its decentralized assortment‐enhancing effects) cannot operate to enforce and favor cooperators when there is commitment to reproduction as an organism or superorganism.

Human societies do not exhibit the same transition in individuality that we see in multicellularity or eusocial insect colonies, but we do see very large‐scale cooperat‐ion and policing of cheaters in human societies. Are the challenges and opportunities in scaling up human cooperation similar to these other systems? We certainly have some of the same opportunities to benefit from cooperation including the ability to pool risk through sharing, divide labor and capitalize on economies of scale. We also encounter some of the same challenges in creating and maintaining a high quality shared environment (take, for example, the tragedy of the commons) and keeping our resource use and reproductive rate within the limits of our carrying capacity. This is true for both local resource availability in small‐scale societies and for global capacity in large‐scale global society.

Thus, we can ask whether humans solve these problems through cheater suppression and committing to cooperation in ways that parallel multicellular bodies and eusocial insect colonies. There are a few lines of work in human evolution and behavior that are relevant to this question. The ability to detect cheating has been argued to be a central component of human social cognition (Cosmides and Tooby 1992, 2005) and across societies, individuals are often willing to incur costs to punish cheaters (Henrich et al. 2006). Interestingly, it has also been argued that one of the factors that allowed humans to scale up to larger societies was the internal regulation of behavior based on supernatural beliefs (Johnson 2005; Marlowe 2009; Henrich et al. 2010; Schloss and Murray 2011). This has interesting parallels to the capacities of cells in multicellular bodies to regulate their resource use, proliferation rate, and other potential sources of cheating through internal regulatory systems.

In smaller scale human interactions, commitment is one tool that can facilitate cooperation (Frank 1988; Nesse 2001). This commitment can be emotionally instantiated in the case of bonding and attachment to family members, mates, or friends that facilitates sharing resources and working toward shared goals. Commitment can also take the form of cultural and institutional systems that constrain behavior and outside options in ways that can enhance cooperation. Sometimes, these types of internal and external commitment overlap in interesting ways. For example, pair bonding is an internal emotional commitment that enhances affiliative behavior, and marriage can serve as an external commitment device that creates costs of leaving and lowers the risk associated with cooperation and investment in shared goals (e.g., raising offspring). The osotua system used by the Maasai is an another example of a hybrid commitment system that appears to involve both emotional engagement with the osotua partner (i.e., genuine concern for their wellbeing) and a cultural framework that makes violations of the osotua system inconceivable (Cronk 2007).

Interestingly, the 3Rs of osotua (respect, restraint and responsibility) correspond in many ways to the ‘expectations’ that multicellular bodies have for the behavior of cells: restrained reproduction and resource use, respect for signals from neighboring cells and the environment, and responsibility to perform required tasks and maintain the shared environment. When cells fail to follow these 3Rs, their neighbors may stop sending survival signals (necessary to prevent cell death). If that fails, they are often targeted for destruction by the immune system.

## Open questions and reflections

### Should we expand our definition of kinship?

In evolutionary biology, we tend to define kinship as genetic relatedness, but this definition is much narrower than definitions used in other disciplines. In the discipline of history, kinship is considered a much broader term encompassing close relationships in which there was some form of inheritance and/or restriction on marriage (Sabean et al. 2007). In anthropology, ‘fictive kinship,’ or the use of kin terms with individuals not closely related is understood to play an important role in cooperation and the establishment of relationships (Cronk and Gerkey 2009). Religious organizations, both historical and modern, use ‘spiritual kinship’ (often in the form of godparent/child relationships) as a way to establish close relationships that often involve gifts and resource transfers. Given the role of these other notions of kinships in human history and modern life, why do we usually define kinship so narrowly in evolutionary biology?

Hamilton's rule, the foundation of inclusive fitness theory, takes *r*, the coefficient of relatedness (see Box 1). But this same rule can use any form of positive assortment as *r*. This rule holds whether *r* refers to genetic relatedness by descent, genetic relatedness not through descent or even just behavioral assortment of cooperators (including individuals of different species) (Fletcher and Doebeli 2009). Any positive fitness interdependence of individuals with one another can provide the material upon which selection for cooperation can act according to Hamilton's rule. So when individuals are dependent on one another through some form of shared fate, this can be functionally identical to a genetic kinship in terms of the strength of selection on cooperation (see Box 1).

This fitness interdependence approach is similar to Roberts’ ‘stakeholder model,’ where individuals have a stake in the wellbeing of others for reasons including but not limited to genetic relatedness (Roberts 2005). This stakeholder model is a way to represent a variety of forms of fitness interdependence including those that arise from genetic relatedness, from repeated interactions (e.g., reciprocity) and even other forms of interdependence such as shared fate. This approach also can encompass scenarios in which individuals have greater stake in another than themselves (which may sometimes be the case for postreproductive individuals) or the negative fitness interdependence that can occur in competitive context including even spiteful scenarios (Roberts 2005).

The stake individuals have in one another's wellbeing is often dependent on the context (e.g., one environment may reward competition, and another, cooperation). In large and complex groups, cooperative and competitive options often exist simultaneously and individuals also often have the ability to decide between taking a cooperative and competitive approach, which will influence whether the stake is positive or negative. Making the decision to choose a cooperative option can be made more viable through certain mechanisms. For example, the decision to enter into a commitment that binds fates together will increase the stake that individuals have in one another. In systems, such as multicellular bodies, the stakes of cell are bound together through the costly commitment to give up independent modes of reproduction and reproduce only through the germ line. In complex human societies, collective costly signaling of having high stake in the wellbeing of partners or group members could lead to a ratcheting to very higher levels.

### Can humans detect fitness interdependence?

This raises the question of whether humans have the capacity to detect situations in which we are highly fitness interdependent with others, that is, when we are in the same figurative boat, and conditionally cooperate in those situations. Common knowledge regarding positive fitness interdependence should make cooperation even more likely. If both individuals know that they need to rely on one another to survive and succeed, it makes cooperation a less risky choice for both. How might such common knowledge be established between individuals who have positive fitness interdependence? One possibility, of course, is the use of kinship terminology. However, this use of language can be exploited by individuals looking to take advantage(Qirko 2011). Thus, the use of kin terminology can be ‘cheap talk,’ that is, not a costly enough (or reliable enough) signal to make it worth taking a risk of cooperation if the stakes are high.

How might individuals signal common knowledge about fitness interdependence in a more reliable way? Costly signals such as rituals and public commitments may be more reliable methods for mutually signaling positive fitness interdependence. Many scholars have proposed that religion and endorsement of sacred values may provide ways for individuals to signal commitment and cooperative intentions (Irons 2001; Sosis and Alcorta 2003; Henrich 2009; Atran and Henrich 2010; Bulbulia 2012; Soler 2012).

More private signals such as mutual entrainment might also serve to create common knowledge about cooperative intentions. Social entrainment involves the reception and processing of rhythmic signals, followed by the generation of rhythmic signals based on the input, which is then processed by the social other (Phillips‐Silver et al. 2010). This capacity for entrainment is what enables us to coordinate rhythmically to produce collective vocalizations, music, and dance. This kind of intense attention and allocation of mental bandwidth to interaction may serve as a reliable signal of willingness to coordinate toward meeting shared goals. Engaging in coordinated vocalization, music, and dance production might be more likely among individuals who perceive positive fitness interdependence or are motivated to establish common knowledge to that effect.

These examples raise the broader question of whether we have the capacity to detect genuine commitment (i.e., cooperative intent) and then behaviorally assort on the basis of it (Aktipis 2000). Social selection (where individuals choose who to have fitness relevant interactions with) could favor both mechanisms for signaling of commitment and mechanisms to accurately detect genuine commitment. This may have led to a signaling arms race on the parts of both signalers and receivers contributing to the complexity of social signals, with the production and detection of vocalizations such as genuine laughter (Bryant 2012; Bryant and Aktipis 2014) and the collective production of music (Hagen and Bryant 2003) and dance (Phillips‐Silver et al. 2010). As a dance instructor during college and graduate school, I was always struck by how powerful a tool dance was for establishing trust and bonds among new acquaintances. I suspect that this may generalize to other domains of rhythmic coordination. Some literature suggests that the act of singing together in karaoke has become an important component of building relationship trust among Taiwanese businessmen (Holt and Chang 2009), suggesting that entrainment may function as a fairly reliable cue of cooperative intent in some modern contexts. Laughter may also be a powerful signal of cooperative intent: the very act of laughing makes us physically weak, making it a genuine and costly way to signal that one is not a threat (Bryant and Aktipis 2014).

What other proximate cues might humans use to assess positive fitness interdependence, or ‘extended kinship’? We know from the incest avoidance literature that living in the same home with someone as a child is a proximate cue to kinship, as is witnessing nursing occurring from the focal individual's mother (Lieberman et al. 2007). But there are other cues that might have been reliably associated with genetic relatedness that could potentially be co‐opted for assessing positive fitness interdependence more generally.

The act of eating together, especially from a ‘shared table’ could be a candidate. Eating together is a central important part of what makes people feel like family. Even within families, eating together fosters feelings of closeness and intimacy. With friends and acquaintances, eating together at a shared table from a common pot makes us all feel more comfortable and trusting. Regularly consuming and sharing food together without aggression or competition over these resources may contribute to our feelings of affiliation and ‘extended kinship’ with others because it is a reliable signal of cooperative intent and having a stake in each other's well‐being. Eating together with an attitude of gratitude seems particularly effective at eliciting positive feelings, at least in our home. The centrality of eating together in human social life and cooperation is an anecdotal fact, but it has also been documented by social psychologists (Argyle 2013) and is implicit in much of the anthropological literature on food sharing (Winterhalder 1986). Whether the act of eating together enhances trust and cooperation is an open question, but the fact that negotiation guides advise that sharing meals is an important contributor to successful outcomes (Graham and Lam 2003; Bernard 2009) is certainly suggestive. Preliminary work at our Malpai field site in southern Arizona and New Mexico suggests that eating meals together is an important part of the need‐based transfer systems.

### Parental investment at the intersection of cooperation and conflict

Parental investment is likely to be the original need‐based transfer, and the language of ‘osotua’ is consistent with this notion: the literal translation is umbilical cord, metaphorically referring to the unidirectional transfer of resources from mother to child. Amy Boddy, who is at the moment a postdoc in my laboratory, has started a research project looking at maternal–fetal interactions themselves from a need‐based transfer perspective. Mothers transfer resources to their offspring based largely on offspring need during gestation (Sibley et al. 2010) and lactation (Daly and Hartmann 1995). However, despite high levels of parental investment in humans, there is still conflict between the mother and offspring over the exact allocation of resources (Haig 1993). Maternal and fetal systems are therefore likely to have cheater detection mechanisms of some type for detecting violations of need‐based transfer rules in resource allocation. Dr. Boddy, myself and our other collaborators often draw on our own experiences as investing parents to guide our broader thinking and develop specific predictions about mother–offspring need‐based transfers. Women, especially mothers, can bring a unique and valuable perspective to the topic of mother–offspring cooperation and conflict, a research topic that has been largely dominated by the sex that is a bystander to the process.

Maternal–offspring conflict illustrates how high levels of conflict often exist among kin. It also calls our attention to the fact that cheater detection systems may be just as necessary for interactions among genetic kin as they are in interactions among unrelated individuals. If need‐based transfers among kin required cheater detection mechanisms (e.g., for offspring who ask when not in need or parents who do not give when able) and effective responses (e.g., punishing or recalibrating behavior in other ways), then these same kin‐based cheater detection systems could also have been co‐opted for detecting cheating in interactions among nonrelatives.

Interestingly, on the other extreme, kin recognition is not necessary for effective parental investment in certain ecological conditions. My models have shown that high rates of correctly directed parental investment can occur when mobility and sociality are low and parental investment occurs over a short period of time (Aktipis and Fernandez‐Duque 2011). On the other hand in species with high mobility, high sociality, and extended periods of investment, it is necessary to have mechanisms for identifying kin to ensure selective resource transfer only to offspring or other relatives.

Over evolutionary time, kin detection mechanisms such as these may have become more generalized to identify a larger class of individuals: those with whom we have shared fates or other forms of interdependent fitness. The results of this model (Aktipis and Fernandez‐Duque 2011) therefore predict that the emergence and generalization of kin detection (to situations in which there is positive fitness interdependence) should be more likely to evolve in species that already have high levels of parental investment, high mobility, and high sociality, for example, in humans.

### How can we use cooperation theory to understand cancer and improve treatment?

Recently, my colleagues and I published a review of cancer across life. We noted how cancer cells ‘cheat’ in the five foundations of multicellular cooperation: proliferation inhibition, controlled cell death, division of labor, resource allocation, and extracellular environment maintenance (Aktipis et al. 2015). We also offered that in advanced stages of cancer there may be a re‐activation or re‐evolution of foundations of multicellular cooperation, in the service of the colony of cancer cells rather than the multicellular body.

Selection for these colony‐level cooperative phenotypes may even be enhanced by Walk Away dynamics, with cells leaving uncooperative clusters and staying in more cooperative ones. There might therefore be selection among colonies favoring cancer cells that produce public goods (e.g., growth factors, angiogenic signals) or otherwise enhance the microenvironment quality from the perspective of the cancer cells (Schiffman et al. in press).

Selection at the colony level could also lead to reproductive division of labor, with only some cells maintaining the capacity to proliferate indefinitely and other cells constraining proliferation and instead contributing to the fitness of the indefinitely proliferating cell from which they are derived. My colleagues and I have shown that this ‘protomulticellularity hypothesis’ is one potential explanation for the existence of so‐called nonstem cells in neoplasms (Sprouffske et al. 2012) and it is in many ways analogous to the collective phenotypes of many social insect colonies.

Cancer is a fascinating subject to study as a cooperation theorist, and it is also an area where evolutionary biology and cooperation theory have much to contribute. My colleagues and I are now applying the foundations of multicellular cooperation framework for cancer to develop assays of cooperation and cheating that could be used in the clinic to guide treatment and risk stratification (Aktipis et al. 2015). We are also applying life‐history theory at both the cellular (Aktipis et al. 2013) and organismal (Boddy et al. 2015) levels to understand cancer susceptibility and develop treatment algorithms that take into account the evolutionary dynamics of neoplasms. We have shown that reproductive competition can reduce the viability of cancer suppression (Boddy et al. 2015; Brown and Aktipis 2015) and that inclusive fitness effects can select for cancer suppression in old age in species with high levels of parental care, grandparental care, and cooperative breeding systems (Brown and Aktipis 2015).

Cooperation and evolutionary approaches not only have a lot to teach us about cancer, but cancer also has a great deal to teach us about the evolution of multicellularity and cooperation. Multicellular bodies are amazing examples of massively large‐scale cooperation. They are possible because conflict among the component parts has been suppressed at many different levels including the cell level and the level of selfish genetic elements. One of the ways in which multicellular bodies suppress this competition is through actually suppressing and slowing the process of somatic evolution itself. DNA repair lowers the mutation rate, and having only a subset of cells indefinitely proliferate lowers the effective population size, thus slowing the rate of evolution. Also, many aspects of tissue architecture serve to suppress the evolutionary process that might otherwise select among cells and eventually lead to neoplastic growth and cancer (Cairns 1975).

The high levels of cooperation we see in multicellular bodies are not a sign of the absence of conflict, but rather the effective suppression of conflict and cheating. Any sufficiently large and long‐lived cooperative system must suppress conflict that inevitably arises from the myriad close associations of its component parts. This suggests that cancer suppression may have some important things to teach us about human social life and how to solve some of the challenges associated with successfully expanding cooperation to large‐scale systems.

### Can we leverage cooperation theory for institutional design?

Humans develop many large‐scale systems that require complex cooperation and monitoring of potential cheating. Cooperation theory can offer some potential solutions to the problems that arise in large‐scale systems. Here, I discuss two possible applications: using positive assortment principles in the peer review process and applying need‐based transfers to the problem of insuring in the face of radical uncertainty.

#### Positive assortment for peer review

We know from both theoretical and empirical work that cooperation is enhanced when cooperators can preferentially interact with one another. Several years ago, my colleague Sharon Thompson‐Schill and I proposed that this first principle of positive assortment could be applied to the peer review process (Aktipis and Thompson‐Schill 2010). We suggested that reviewers could be given a score based on speed of reviewing, rate of reviewing, or other priorities of the journal editor. Authors would then be paired with reviewers who have similar scores. This could increase the speed of reviewing and decrease the burden on reviewers at no financial cost. This solution to the problem of the ‘tragedy of the commons’ of peer review (Hochberg et al. 2009) has not been taken up yet by any journals (to our knowledge), but in our conversations with journal editors, many have been intrigued.

#### Need‐based transfers for decentralized insurance

On a much larger scale, the principle of need‐based transfers could potentially be leveraged in institutional design in ways can provide solutions some of the problems that arise from our living in an increasingly uncertain world. Sharing in times of need is an ancient human solution for managing the risk associated with living in a volatile environment (Winterhalder 1986). In modern life, we now often choose to invest in insurance to protect us from potential shocks. However, not all negative events can be insured against because some are so rare that they cannot be assigned a probability. This kind of uncertainty, sometimes called radical uncertainty or Knightian uncertainty (Knight 1921), poses a problem for insurance systems because it cannot be quantified using standard actuarial approaches. Need‐based transfer relationships, like the osotua relationships among the Maasai, do not require a quantification of all risks to function as an effective decentralized insurance system so they might offer solutions to the problems associated with radical uncertainty.

In need‐based transfer systems, individuals help each other in times of need, up to their ability to help without putting themselves at risk. This functions as a system of limited risk pooling that can provide risk mitigation for the entire network without making the whole network vulnerable. Need‐based transfer relationships may be able to insure against radical uncertainty in some cases because they do not require exact assessment of the probabilities of negative events. When individuals recognize their reliance on one another and their interdependence (i.e., when *s* is high, see Box 1), their willingness to make commitments to mutual aid may increase. Our global society is becoming increasingly interdependent, and we are all living in a more and more uncertain world. This suggests that need‐based transfer systems can perhaps be leveraged to expand our capacity to deal with risk and uncertainty as our world changes more and more rapidly. As part of The Human Generosity Project, we are working with the Decision Center for a Desert City at ASU and the Extension Disaster Recovery Education Network at Cornell to translate need‐based transfers to modern resource management and disaster recovery challenges through workshops and discussions with policymakers.

## Conclusions

As humans, we seem compelled both to understand human nature and to discuss our views about human nature with one another. This debate about whether human nature is essentially cooperative or competitive goes back to early philosophical writings and has divided people for centuries. Is it much of a surprise then, that it divides much of the cooperation theory community today? Yes and no. ‘No,’ because this questions of whether altruism really exists cuts to the core of our beliefs about human nature and is therefore deeply important to scientists, philosophers, and every person trying to navigate complex human social life. And ‘yes’, it is surprising that it continues to be debated so vehemently because we now have mathematical and computational models that now tell us that cooperation and competition can and do coexist in most systems. These models allow us to investigate the fundamental dynamics that shape cooperation and conflict, and answer questions about the nature of social life that we have never before been able to examine with such power. Why, then, do we continue to debate these topics, even with the tools to answer many of these questions?

This may be largely a psychological and sociological question, related to issues like our in‐group/out‐group psychology, how we conceptualize kinship, and even our folk psychology of parenting. In our own social lives, we often focus on either the aligned interests or the conflicting interests we have with one another, rather than acknowledging the complexities. This might indeed be adaptive for many situations: It can serve to organize goals and behavior and might also signal our intent to other parties in ways that improve outcomes. However, our evolved psychology of cooperation and conflict might sometimes be a barrier to understanding the nuances of complex social situations in which goals exist at multiple levels of organization and even within a single relationship, where high levels of conflict and cooperation might coexist.

This is perhaps nowhere more clear than in familial relationships. Large family events are often characterized by conflict as much as they are by cooperation, an anecdotal fact that has received surprisingly little attention from academics interested in family relationships. But our experience tells us that conflict exists among genetic kin, just as it does among nonrelatives, and models of kin conflict tell us that sometimes resource competition is even more likely among genetic kin than nonrelatives because of limited dispersal (West et al. 2002). This means that even among genetic kin it is necessary to suppress conflict and cheating to create large‐scale and long‐lived aggregations. The potential for cooperation, too, exists among individuals of all types, whether genetic kin, nonrelatives or even individuals of another species. Perhaps expanding our definition of kinship in the biological sciences or simply using the broader term of ‘positive fitness interdependence’ could help us to see past our intuition that genetic relatives are a privileged class of individuals when it comes to the benefits of cooperating.

Many of my experiences in life and science have led me to the same conclusion: Conflict and cooperation are not two opposites, but can and often do coexist. In fact, they often go hand‐in‐hand when individuals are in close association with one another. Whether we are considering two siblings, a married couple, or academics working closely together on a project, we see the same general principle: More interaction means more opportunities for both conflict and cooperation. Or in the language of evolutionary cooperation theory, close associations foster both positive and negative fitness interdependence.

How, then, can we effectively navigate relationships characterized by both opportunities for cooperation and the challenges associated with conflict? Perhaps the advice to eat, laugh, and dance together is not just a trite nicety, but instead an idea well worth testing in the laboratory and in our own lives. I would offer from my own experience and cultural background (which is equal parts Greek Islander and Viennese culture lover), that food, music, dance, and other types of shared exuberance form much of the fabric of trust and positive mutual engagement within families and beyond. Whether it is with genetic kin, close friends who feel like family, or new acquaintances who you are just getting to know, sharing a meal and a good laugh may go a long way toward helping navigate the complexities of human social living.

## Note

1 My work on need‐based transfers is in collaboration with Lee Cronk, an Anthropologist at Rutgers University. We first began using the term ‘need‐based transfers’ after Daniel Hruschka suggested that it was the appropriate term to describe the algorithm we were studying in human sharing systems.
